# Preliminary study on the application of renal ultrasonography radiomics in the classification of glomerulopathy

**DOI:** 10.1186/s12880-021-00647-8

**Published:** 2021-07-23

**Authors:** Lijie Zhang, Zhengguang Chen, Lei Feng, Liwei Guo, Dong Liu, Jinjin Hai, Kai Qiao, Jian Chen, Bin Yan, Genyang Cheng

**Affiliations:** 1 Department of Nephrology, The First Affiliated Hospital of Zhengzhou University, Zhengzhou, Henan China; 2 Department of Ultrasound, The First Affiliated Hospital of Zhengzhou University, Zhengzhou, Henan China; 3 Department of Nephrology, Zhengzhou Ninth People’s Hospital, Zhengzhou, Henan China; 4 PLA Strategy Support Force Information Engineering University, Zhengzhou, Henan China

**Keywords:** Radiomics, Ultrasonography, Histologic classification, IgA nephropathy, Membranous nephropathy

## Abstract

**Background:**

The aim of this study was to investigate the potential use of renal ultrasonography radiomics features in the histologic classification of glomerulopathy.

**Methods:**

A total of 623 renal ultrasound images from 46 membranous nephropathy (MN) and 22 IgA nephropathy patients were collected. The cases and images were divided into a training group (51 cases with 470 images) and a test group (17 cases with 153 images). A total of 180 dimensional features were designed and extracted from the renal parenchyma in the ultrasound images. Least absolute shrinkage and selection operator (LASSO) logistic regression was then applied to these normalized radiomics features to select the features with the highest correlations. Four machine learning classifiers, including logistic regression, a support vector machine (SVM), a random forest, and a K-nearest neighbour classifier, were deployed for the classification of MN and IgA nephropathy. Subsequently, the results were assessed according to accuracy and receiver operating characteristic (ROC) curves.

**Results:**

Patients with MN were older than patients with IgA nephropathy. MN primarily manifested in patients as nephrotic syndrome, whereas IgA nephropathy presented mainly as nephritic syndrome. Analysis of the classification performance of the four classifiers for IgA nephropathy and MN revealed that the random forest achieved the highest area under the ROC curve (AUC) (0.7639) and the highest specificity (0.8750). However, logistic regression attained the highest accuracy (0.7647) and the highest sensitivity (0.8889).

**Conclusions:**

Quantitative radiomics imaging features extracted from digital renal ultrasound are fully capable of distinguishing IgA nephropathy from MN. Radiomics analysis, a non-invasive method, is helpful for histological classification of glomerulopathy.

**Supplementary Information:**

The online version contains supplementary material available at 10.1186/s12880-021-00647-8.

## Background

Kidney disease is thought to be a substantial worldwide clinical and public health problem [1]. In China, the overall prevalence of chronic kidney disease (CKD) is 10.8% [1]. CKD is a “silent killer” due to its insidious onset and slow progression to end-stage renal disease. Early diagnoses and rational therapeutic strategies are critical for controlling the progression and improving the prognoses of patients with CKD. Primary glomerulopathy is the most common cause of CKD. It includes several pathological types, such as IgA nephropathy, membranous nephropathy (MN), minimal-change glomerulopathy and focal segmental glomerulosclerosis. Epidemiological investigations have shown that IgA nephropathy and MN are the most common pathological types [2]. Accurate classification of glomerulopathy is very important for both treatment optimization and prognosis prediction. For example, according to KDIGO guidelines [3], for patients at high risk of disease progression, steroids should be considered for IgA nephropathy (recommended level: 2B). However, the use of immunosuppressants is controversial. Unlike the recommended treatment for IgA nephropathy, steroids combined with immunosuppressants are recommended for patients with MN (recommended level: 1B).

The diagnosis and classification of primary glomerulopathy are based mainly on clinical manifestations, laboratory examinations and renal pathological diagnosis. However, clinical manifestations and laboratory test results usually lack specificity. Pathology depending on renal biopsy is the gold standard for accurate diagnosis and classification of glomerulopathy. However, the examination is an invasive test with many complications and contraindications, which limits its use in clinical settings [4]. In addition, many hospitals with limited resources lack adequate facilities to perform kidney biopsies. Therefore, a non-invasive test that can replace renal biopsy is urgently needed.

Renal ultrasound is widely used in the clinical examination of patients with nephropathy due to its simple operation, rapidity, and low cost. Sonography of the kidneys provides information on kidney size, kidney thickness, and renal cortex echogenicity. This technique is frequently employed during the evaluation of renal failure degree, and the findings often provide the bases for decisions about whether a renal biopsy should be performed. In glomerulonephritis, renal ultrasound shows parenchymal diffuse echo changes. Additionally, the relationship between the degree of renal cortical echogenicity and renal histological change has been demonstrated [5]. Several studies have also shown that the degree of echogenicity of the renal cortex might reflect the severity of glomerular sclerosis/crescent formation, tubular atrophy, and interstitial inflammation/fibrosis [6]. However, it is not clear whether the specific sonographic appearance is related to the pathological classification. Fortunately, the achievements of radiomics in ultrasonography-based diagnosis have enabled a breakthrough.

Radiomics is a newly developing technique that extraction and analysis of a large number of advanced quantitative image features from radiographic images, such as computed tomography (CT), magnetic resonance imaging (MRI), and positron emission tomography (PET) features [7]. Multiple studies have shown that radiomics can automatically extract and analyse histogram, texture, and shape information from imaging data that might not be evident to the naked eye and convert it to quantitative and minable high-dimensional data [8, 9]. The technique has achieved impressive success in tumour diagnosis, accurate classification, treatment response assessment, and prognosis [10]. Ultrasound-derived quantitative features using radiomics have been used to discriminate malignant tumours from benign tumours in thyroid [11] and breast [12] tissues. However, radiomics has not yet been applied to kidney disease. Since the degree of renal cortical echogenicity can reflect the extent of renal parenchymal lesions, we attempted to apply radiomics to renal ultrasound and extract renal ultrasonographic features to aid the histologic classification of glomerulopathy.

In this study, we analysed the potential application value of radiomics based on renal ultrasound images in the classification of IgA nephropathy and MN. The method in this study will provide an unprecedented opportunity to improve nephropathy diagnosis and decision support in nephropathy treatment. To the best of our knowledge, this is the first study to analyse the application of radiomics features in renal ultrasound images for the classification of IgA nephropathy.

## Methods

### Patient characteristics

This study was a cross-sectional study. The study was carried out in the Department of Nephrology, First Affiliated Hospital of Zhengzhou University, from July 2019 to December 2019.

Inclusion criteria: 1. Patients who were adults and who had undergone renal biopsy for diagnosis of the cause of proteinuria, haematuria, azotaemia, and/or renal insufficiency; 2. patients whose pathological results confirmed IgA nephropathy or MN; and 3. patients not administered glucocorticoids or immunosuppressants before renal biopsy.

Exclusion criteria: 1. Non-MN and non-IgA nephropathy patients; 2. patients whose disease co-existed with other pathological types (e.g., patients with MN coexisting with IgA nephropathy); 3. patients who had previous undergone renal biopsy; 4. renal transplant recipients; and 5. patients with kidney stones, renal cysts, or tumours.

### Clinical data collection

For each patient, demographic data, including age and sex, were recorded. Blood pressure was measured with a sphygmomanometer at 8:00 am by qualified physicians. Venous blood samples were collected from all subjects in the fasting state at admission before renal biopsy. Complete blood counts and routine urinary and biochemical analyses were performed with standard laboratory methods in the clinical laboratory. The estimated glomerular filtration rate (eGFR) was calculated with the CKD Epidemiology Collaboration (CKD-EPI) equation [13]. Twenty-four-hour urine was collected, and urinary protein was quantified.

### Ultrasound measurement and image capture

1. *Instrument* Ultrasound testing was performed with a Mindray Resona 7 colour Doppler ultrasound with a convex array probe of 3.5 MHz.
2. *Examination Methods* The test was performed on the day before renal biopsy by the same operator for all patients. The subjects were usually positioned in a prone position. The longitudinal diameter and renal parenchymal thickness of the right kidney were measured in the maximum long-axis and transverse views. Ultrasound images of the kidney at different cross-sections were acquired. All images were collected with the same model of ultrasound machine with the same parameters and saved as DICOM data to ensure the consistency of images.
3. *Grouping* Ultimately, 623 renal ultrasound images from 46 MN patients and 22 IgA nephropathy patients were obtained. To reduce bias, these images were randomly divided into a training data set (51 cases with 470 images) and a validation data set (17 cases with 153 images) based on the assumption that all data exhibited the same data distribution.

### Renal biopsy

For all patients, renal biopsy was performed after ultrasonographic assessment. It was performed under local anaesthesia with lidocaine, and 16- or 18-G biopsy needles were used. The renal biopsy specimens were preserved in 10% formaldehyde, embedded in paraffin, sectioned at 6–8 µm, and subsequently stained with haematoxylin–eosin, trichrome Gomori, and periodic acid-Schiff. Finally, the samples were assessed by at least two pathologists via light microscopy and electron microscopy.

### Radiomics analysis

In the kidney ultrasound images, the renal parenchyma in each slice was manually segmented as the region of interest (ROI). Subsequently, we designed and extracted a total of 180 dimensional radiomics features based on the ROIs. Next, the least absolute shrinkage and selection operator (LASSO) logistic regression algorithm was used to reduce the dimensionality of the extracted radiomics features and select the most related features. Four classifiers (logistic regression, a support vector machine (SVM), a random forest, and the K-nearest neighbour (KNN) method) were used to distinguish the pathological types of glomerulopathy. The diagnostic performance was evaluated according to classification accuracy, the area under the receiver operating characteristic (ROC) curve (AUC), sensitivity, and specificity. The analysis details are given below.

### Ultrasound image segmentation

**Fig. 1:**
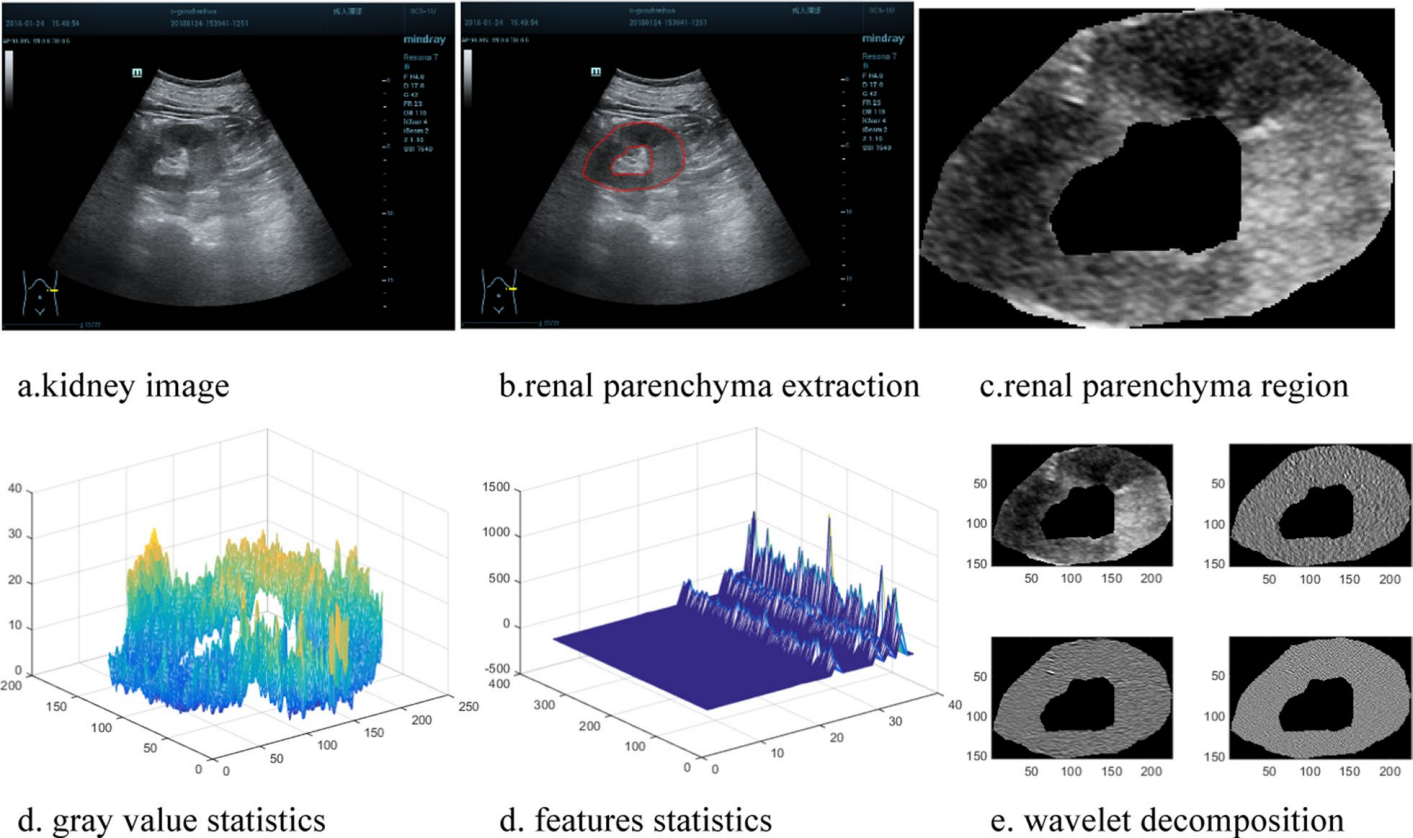
The feature analysis workflow of kidney image. **a**–**c** are the extracting route of the renal parenchyma region. **a** kidney image; **b** renal parenchyma extraction; **c** renal parenchyma region; **d**–**f** are the radiomics feature computation. **d** gray value statistics; **e** features statistics; **f** wavelet decomposition

Since the ultrasound images of the kidneys contained non-renal parenchymal parts, which might have confused the classifiers during discrimination of nephropathy, the renal parenchyma was first extracted to ensure that the model focused on that region rather than the other parts of each image. This step was beneficial for nephropathy classification. We delineated the renal parenchyma in every slice (through manual segmentation). The first row in Fig. 1 shows the renal parenchyma extraction process. The renal parenchyma in ultrasound images often appeared in the annulus, which is delineated in Fig. 1b. To reduce errors, the renal parenchyma in all renal ultrasound images was outlined using a labelling tool that we developed and modified with two other professional radiologists. In cases of disagreement, the final renal labelling was decided through discussion among these two radiologists and their colleagues.

### Radiomics feature extraction and selection

Based on the delineated renal parenchyma regions, a total of 180 dimensional features were designed and extracted. These features contained 14 dimensional gray-level features, 9 dimensional texture features derived from the gray-level co-occurrence matrix (GLCM) [14], 13 dimensional texture features derived from the gray-level run-length matrix (GLRLM) [15, 16], and 144 dimensional gray-level and texture features based on sub-images obtained by two-dimensional discrete wavelet decomposition at a single level (Table 1). All radiomics features mentioned were examined in every kidney image slice with the MATLAB programming platform (version R2014b). All the radiomics features were normalized to a 0–1 scale in our experiments. Considering that high-dimensional features could easily lead to overfitting of the learning algorithm and that some features might have no impact on the task of nephropathy classification, we employed supervised LASSO regression to select the most related features and to decrease the dimensionality of the features [17]. LASSO regression estimated a vector of regression coefficients by minimizing the residual sum of squares subject to a constraint on the L1-norm of the coefficient vector. Because our focused nephropathy classification was actually binary classification, the linear model was replaced in LASSO regression with logistic regression. LASSO logistic regression was used for nephropathy classification, which was further performed in R (version 3.5.1; open-source software, https://www.r-project.org/).

**Table 1:** Extracted Radiomics features in our paper

Feature type	Feature name
Gray-level	Energy Entropy Kurtosis Maximum Mean Mean absolute deviation Mediam Minimum Rang Root mean square Skewness Standard deviation Uniformity Variance
GLCM	Energy Contrast Entropy Homogeneity Correlation Variance Sum average Dissimilarity Autocorrelation
GLRLM	Short Run Emphasis Long Run Emphasis Gray-Level Nonuniformity Run-Length Nonuniformity Run Percentage Low Gray-Level Run Emphasis High Gray-Level Run Emphasis Short Run Low Gray-Level Emphasis Short Run High Gray-Level Emphasis Long Run Low Gray-Level Emphasis Long Run High Gray-Level Emphasis Gray-Level Variance Run-Length Variance
Wavelet	Gray-level and texture features calculated on four non-decimated 2-D wavelet decomposition

### Classification algorithms

To confirm the relationships of the quantitative radiomics imaging features with glomerulopathy, the selected radiomics features were used as the inputs for four machine learning algorithms, namely, logistic regression, an SVM algorithm, a random forest and a KNN algorithm, to differentiate MN and IgA nephropathy on ultrasound images. A non-invasive radiomics signature prediction model was constructed. These four machine learning algorithms were implemented with the sklearn package (version 0.19.1; https://scikit-learn.org/) in Python.

### Statistical analysis

The data in this study with normal distributions are shown as the means ± standard deviations (SDs), whereas data with non-normal distributions are reported as the median and interquartile range. Independent-sample t-tests and Mann–Whitney U tests were applied for statistical analysis. All *p* values are two-sided, and a *p* value of less than 0.05 was considered to indicate significance. The analyses were conducted by using SPSS statistics version 22.0 (IBM Corp., Armonk, NY, USA).

## Results

### Patient characteristics

A total of 68 adult patients were enrolled, including 46 MN and 22 IgA nephropathy patients diagnosed by renal biopsy. The clinical characteristics, biochemical data, and ultrasonographic kidney measurements are summarized in Table 2. Patients with MN were older than those with IgA nephropathy. Total cholesterol, low-density lipoprotein, and 24-h urinary protein levels were significantly higher in patients with MN than in patients with IgA nephropathy (*p* < 0.05). Kidney size was not significantly different between the indicated two groups (*p* > 0.05).

**Table 2:** Comparison of clinical parameters between IgA patients and MN patients

	IgA(n = 22)	MN(n = 46)	P value
Male (%)	12 (40.9%)	30 (65.2%)	0.317
Age (year)	39.29 ± 10.80	49.48 ± 12.62	0.002
SBP (mmHg)	132.57 ± 18.27	133.09 ± 14.92	0.903
DBP (mmHg)	82.76 ± 10.53	88.16 ± 15.59	0.156
Hb (g/L)	121.57 ± 20.35	129.99 ± 17.19	0.084
BUN (mmol/L)	8.06 (3.60, 15.40)	5.86 (2.30, 10.90)	0.141
Cr (mmol/L)	106.14 (53.00, 222.00)	73.19 (36.00, 137.00)	0.361
UA (mmol/L)	378.0 ± 127.47	336.61 ± 112.96	0.186
eGFR (ml/min.1.75m^2^)	73.67 (13.60, 122.74)	92.94 (39.54, 141.08)	0.722
Alb (g/L)	35.42 (13.60, 44.10)	25.01 (15.10, 41.20)	0.316
TC (mmol/L)	4.69 ± 1.12	7.31 ± 2.29	0.000
TG (mmol/L)	1.56 (0.58, 4.85)	2.77 (0.87, 5.89)	0.053
HDL (mmol/L)	1.34 (0.76, 2.86)	1.72 (0.67, 4.28)	0.877
LDL (mmol/L)	2.96 ± 0.90	4.96 ± 2.12	0.000
Hematuria(%)	19 (86.4%)	24 (52%)	0.050
Urinary protein quantitative (g/24 h)	1.38(0.73, 2.30)	4.59 (2.64, 6.42)	0.000
Renal longitudinal diameter (mm)	103.20 (90.00, 130.00)	108.84 (93.00, 130.00)	0.968
Renal parenchyma thickness (mm)	14.73 (10.00, 20.00)	15.35 (12.00, 20.00)	0.508

P < 0.05 has been accepted to be significant. Normal distribution data are presented as means ± SD. Abnormal distribution data are presented as median, 25 percentile, and 75 percentile

*SBP* systolic blood pressure, *DBP* diastolic blood pressure, *Hb* hemoglobin, *TP* total protein, *Alb* albumin, *BUN* blood urea nitrogen, *Cr* creatinine, *UA* uric acid, *TC* total cholesterol, *TG* triglyceride, *HDL* high-density lipoprotein, *LDL* low-density lipoprotein

For analysis, the images in the two groups were divided into two other groups: a test group and a training group. Seventy-two images of 8 patients in the IgA group and 81 images of 9 patients in the MN group were randomly selected as the validation set. The remaining images of each group were included in the training set. The clinical characteristics, biochemical data, and renal ultrasonographic measurements of the training set and validation set for the IgA patients and MN patients are summarized in Table 3. No indicators were significantly different between the training set and validation set for either group.

**Table 3:** Comparison of clinical parameters between the training set and validation set of IgA patients and MN patients

	IgA(n = 22)		MN(n = 46)	
	Training set (n = 14)	Validation set (n = 8)	Training set (n = 37)	Validation set (n = 9)
Age (year)	37.08 ± 9.27	42.88 ± 12.74	49.59 ± 12.48	49.00 ± 13.98
SBP (mmHg)	127.38 ± 6.87	141.00 ± 27.24	131.83 ± 13.40	138.11 ± 20.10
DBP (mmHg)	80.15 ± 6.62	87.00 ± 14.44	87.31 ± 11.92	91.56 ± 26.43
Hb (g/L)	116.42 ± 19.44	129.94 ± 20.16	129.29 ± 17.47	132.87 ± 16.65
BUN (mmol/L)	7.62 (4.00, 15.40)	8.72 (3.60, 14.50)	5.68 (3.20, 10.60)	6.71 (2.30, 10.90)
Cr (mmol/L)	104.65 (53.00, 189.00)	108.25 (53.00, 222.00)	72.27 (36.00, 102.00)	77.45 (36.00, 137.00)
UA (mmol/L)	351.89 (244.00, 624.00)	366.42 (84.00, 538.00)	336.54 ± 117.83	336.89 ± 96.42
eGFR (ml/min.1.75m^2^)	70.93 ± 35.46	69.09 ± 33.54	93.47 ± 19.50	88.99 ± 29.48
Alb (g/L)	36.45 (19.30, 44.10)	33.87 (13.60, 43.8)	24.78 ± 6.76	24.80 ± 6.42
TC (mmol/L)	4.94 (2.61, 6.17)	4.56 (3.24, 5.57)	7.39 ± 2.19	6.97 ± 2.75
TG (mmol/L)	1.54 (0.58, 4.85)	1.60 (0.66, 3.40)	2.90 ± 1.36	2.43 ± 1.33
HDL (mmol/L)	1.29 (0.84, 2.70)	1.41 (0.76, 2.86)	1.49 ± 0.52	2.20 ± 1.26
LDL (mmol/L)	3.21 ± 1.01	2.61 ± 0.59	5.14 ± 2.09	4.18 ± 2.16
Hematuria(%)	11 (79%)	8 (100%)	22 (59%)	2 (22%)
Urinary protein quantitative (g/24 h)	1.58 (0.67, 2.70)	1.20 (0.75, 1.50)	3.90 (2.44, 6.34)	5.62 (4.00, 7.61)
Renal longitudinal diameter (mm)	103.77 ± 10.16	103.25 ± 10.79	108.62 ± 7.51	110.33 ± 14.00
Renal parenchyma thickness (mm)	15.33 (10.00, 20.00)	13.83 (12.00, 16.00)	15.22 (12.00, 20.00)	16.00 (13.00, 19.00)

P < 0.05 has been accepted to be significant. The p values of the above statistical results were all greater than 0.05. Normal distribution data are presented as means ± SD. Abnormal distribution data are presented as median, 25 percentile, and 75 percentile

*SBP* systolic blood pressure, *DBP* diastolic blood pressure, *Hb* hemoglobin, *Alb* albumin, *BUN* blood urea nitrogen, *Cr* creatinine, *UA* uric acid, *TC* total cholesterol, *TG* triglyceride, *HDL* high-density lipoprotein, *LDL* low-density lipoprotein

### Selection of radiomics features

**Fig. 2:**
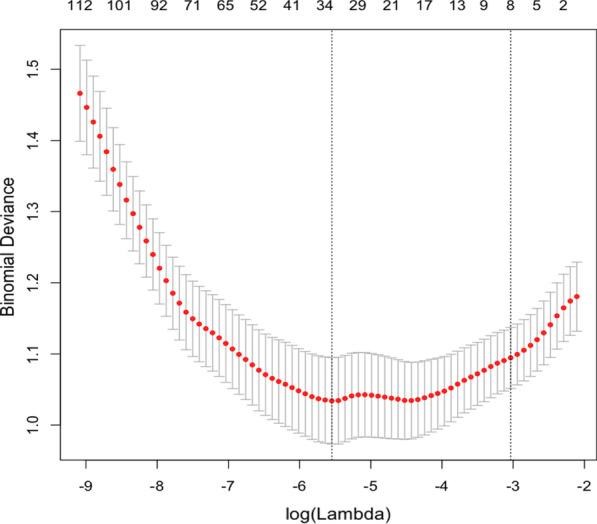
Cross-validated deviance with the number of non-zero coefficients and Lambda fit by LASSO logistic regression

For LASSO logistic regression, we selected an appropriate regularization parameter (λ) according to the cross-validated deviance. The data in Fig. 2 describe the mapping relationship of the cross-validated deviance with non-zero coefficients (that is, the number of selected radiomics features) and the lambda fit by LASSO logistic regression. The top axis is the number of non-zero coefficients. The left vertical dashed line represents the λ providing the smallest cross-validated deviance. Additionally, the right vertical dashed line represents the minimum deviance plus no more than one standard deviation. Collectively, we selected 33 features based on the smallest cross-validated deviance. Table 4 shows the 33 selected radiomics features.

**Table 4:** The selected 33 radiomics features by LASSO logistic regression

Feature type	Feature name
Gray-level	Entropy
	Uniformity
GLCM	Contrast
	Sum average
GLRLM	Short Run Low Gray-Level Emphasis Short Run High Gray-Level Emphasis Long Run Low Gray-Level Emphasis Long Run High Gray-Level Emphasis Gray-Level Variance
Wavelet	Entropy Mediam Contrast Variance Sum average Gray-Level Nonuniformity Run-Length Nonuniformity
	Mediam Energy Correlation Sum average Short Run Emphasis
	Contrast Correlation Gray-Level Nonuniformity Short Run Low Gray-Level Emphasis Run-Length Variance
	Kurtosis Skewness Energy Gray-Level Nonuniformity Short Run Low Gray-Level Emphasis Gray-Level Variance Run-Length Variance

### Nephropathy classification performance

**Fig. 3:**
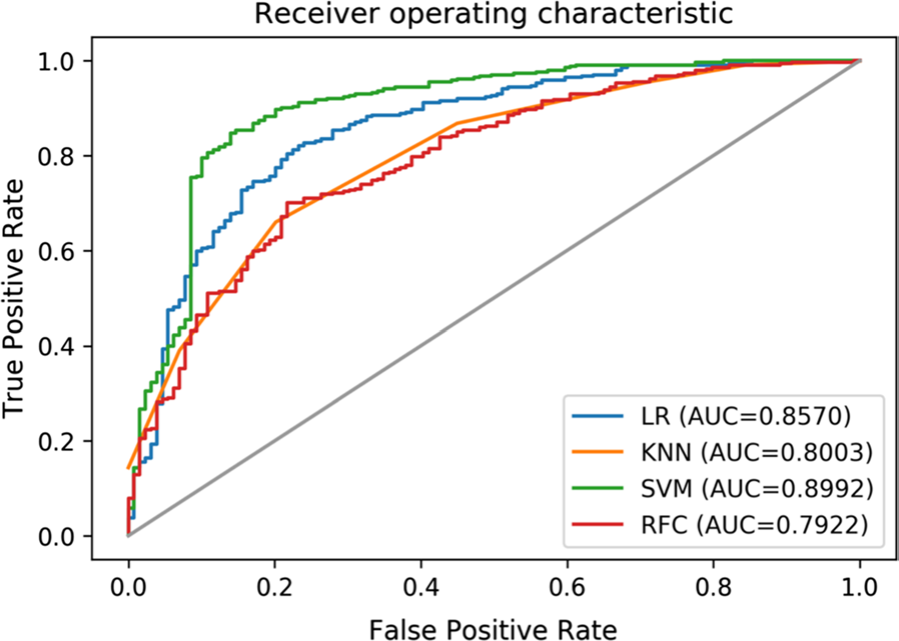
Training ROC curve of different classifiers on image slices

We then evaluated the classification performance of our proposed algorithms for the collected data. Figure 3 shows the training ROC curves of the different classifiers for the image slices, and Table 5 shows the classification performance of the different classifiers for the image slices in the training stage. The accuracy, AUCs, specificity, and sensitivity of the four classifiers based on the selected 33 features for the validation set are shown in Table 6. Since every patient had several ultrasonographic slices and since the nephropathy was identical, we computed the accuracy and AUC values for ultrasonographic slices and patients, respectively. The mean predicted probability of all slices in each patient served as the probability for that patient. The validation performance was slightly lower than the training performance, which was normal, and the difference between the training and validation performance was slight, indicating that the training set was not severely over-fitted. Notably, the feature dimensions and classifier capacity were matched.

**Table 5:** Training classification performance of different classifiers on image slices

	LR	KNN	SVM	RFC
Accuracy	0.8128	0.7723	0.8234	0.7213
AUC	0.8570	0.8003	0.8992	0.7922

**Table 6:** The nephropathy classification performance of four classifiers

Model		Accuracy	AUC	Specificity	sensitivity
Logistic regression	Test set	**0.7647**	0.7500	0.7500	**0.8889**
SVM	Test set	0.7059	0.7222	0.7500	**0.8889**
Random forest	Test set	0.7059	**0.7639**	**0.8750**	0.6667
KNN	Test set	0.5294	0.7361	**0.8750**	0.5556

**Fig. 4:**
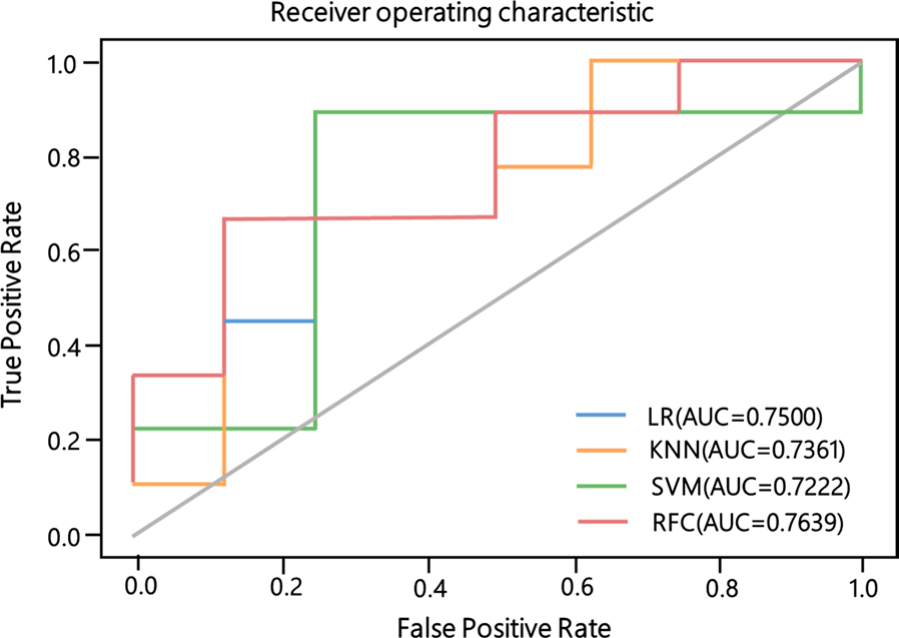
The ROC curve of four classifiers on nephropathy classification

The four classification models all exhibited good performance based on the extracted and selected radiomics features. The AUCs of all models were higher than 0.7 for the test data, and the random forest had the highest AUC at 0.7639. Logistic regression had the highest accuracy at 0.7647, while the KNN method had the lowest accuracy at 0.5294. The random forest and KNN model had the highest specificity (0.8750), suggesting that their ability to recognize IgA nephropathy was strong and that these two classifiers had a lower misdiagnosis rate than the others. Logistic regression and the SVM had the highest sensitivity at 0.8889, suggesting that their ability to recognize MN was strong and that these two classifiers had a lower missed diagnosis rate than the other classifiers. Importantly, the ROC curves shown in Fig. 4 also confirmed the sensitivity and specificity of the different classifiers.

## Discussion

Here, we performed radiomics analysis to quantitatively characterize the ultrasonographic imaging features of IgA nephropathy and MN. Our results revealed that the radiomics-based method exhibited excellent performance for differentiating IgA nephropathy from MN on ultrasound images. To our knowledge, this is the first application of radiomics for prediction of the renal pathologic type of primary glomerulopathy on the basis of renal ultrasound.

Glomerulopathy can be classified according to demographic characteristics (such as age and sex) and clinical manifestations (such as haematuria and urinary protein) [18]. In our study, IgA nephropathy occurred frequently in adolescents. The clinical features were chronic glomerulonephritis characterized by asymptomatic haematuria with urinary protein that generally did not exceed 2.0 g/24 h. In contrast, MN primarily affected middle-aged adults. It often manifested as nephrotic syndrome including significant proteinuria, hypoproteinaemia and hyperlipidaemia. Therefore, the clinical parameters of IgA nephropathy and MN presented in Table 2 were consistent with those in a previous study [18]. However, the accuracy of the predictions was not as high as expected. Li C et al. [19]. trained and validated a machine learning algorithm using data from 222 patients (88 MN, 28 IgA) to build a pathological prediction model for primary nephrotic syndrome. The algorithm identified 17 of 33 variables as contributing strongly to the type of renal pathology. The accuracy of model prediction for MN was 76.1%, whereas the accuracy for IgA nephropathy was only 57.1%. In addition to assessment of clinical manifestations, several other methods are commonly used to distinguish the pathological types of kidney disease, such as renal biopsy and biomarker analysis. Renal biopsy is the gold standard for diagnosis. However, due to the invasiveness of examination and the many contraindications, its clinical application is limited. Importantly, the discovery of biomarkers has improved the accuracy of non-invasive diagnosis of glomerulonephritis. In 2009, podocyte phospholipase A2 receptor (PLA2R) was reported as an antigenic target in autoimmune adult MN. Anti-PLA2R antibodies in serum have high specificity (close to 100%) and sensitivity (70–80%) for diagnosis of primary MN [20]. Notably, the antibody tests can only be used to identify primary MN. Specific biomarkers have not been discovered for IgA nephropathy or other types of glomerular diseases, which limits the clinical application of biomarkers [21].

Renal ultrasound can indicate the shape, blood supply and elasticity of the kidney and is used to evaluate kidney function and pathology [5, 6]. However, this technique has not been used for the pathological classification of glomerulopathy due to technical limitations. Radiomics feature analyses and machine learning algorithms are promising methods for optimization of radiology evaluation and have been shown to perform well for various classification problems and imaging modalities. Radiomics has been used to predict the histological subtypes of renal tumours preoperatively [22]. However, unlike the focal lesions of renal cell carcinoma, glomerular lesions are usually diffuse lesions in both kidneys and are examined by ultrasound, not CT or MRI. This is the first attempt to classify diffuse glomerular lesions by using ultrasound imaging analysis-based radiomics. In this study, a series of 180 dimensional features were extracted from renal ultrasound images of IgA nephropathy and MN to describe renal characteristics. We used a supervised feature selection algorithm (LASSO regression), which differs from several other dimension reduction methods that do not consider the object class, to find the features most related to nephropathy for classification. The highest classification AUC of 0.7639 and the highest specificity of 0.8750 were achieved by the random forest. Logistic regression attained the highest accuracy of 0.7647 and the highest sensitivity of 0.8889. Our model based on ultrasound images was significantly better than a model based on clinical data [19]. Our study demonstrates that biopsy-free prediction of the pathologic type of glomerulopathy based on renal ultrasonography radiomics is feasible and promising.

Based on the LASSO logistic regression coefficients assigned to each feature, the increased weights of the 33 selected features were obvious. The features with the highest weights were as follows: the gray-level variance; the sum average and run-length non-uniformity of 2-dimensional wavelet AA decomposition; the energy of 2-dimensional wavelet AD decomposition; the correlation, gray-level non-uniformity, and short-run low-gray-level emphasis of 2-dimensional wavelet DA decomposition; and the kurtosis, skewness, energy, gray-level non-uniformity, short-run low-gray-level emphasis, gray-level variance, and run-length variance of 2-dimensional wavelet DD decomposition (‘A’ stands for the low-pass filter, and ‘D’ stands for the high-pass filter). We found that the features with the highest weights were texture features based on the GLCM and GLRLM. Texture is an important characteristic for identifying objects or ROIs in an image. Textural features are based on gray-tone spatial dependencies and distributions. The distributions of run lengths and the distributions of gray values of runs are distinct entities. In addition, texture is correlated with image scale, and wavelets are suitable for extracting multi-scale texture features. The renal parenchymas in different ultrasound images have different shapes, scales, and views. Therefore, texture features based on wavelet decomposition are very useful for image recognition, and the aforementioned highest-weighted features also prove this.

The effect of feature selection was also evaluated through comparison of the classification performance between all the radiomics features and selected features. We implemented fivefold cross validation to select 33 features based on the smallest cross-validated deviance. The accuracy of logistic regression and the SVM for the selected features was higher than that for the non-selected features (Additional file 1: Table 1). The AUCs of the logistic regression, random forest and KNN methods for the selected features were also higher than those for the non-selected features. Therefore, feature selection plays a positive role in nephropathy classification because LASSO logistic regression is a supervised algorithm that can select features that are related to nephropathy types. There are also other object optimization criteria in addition to deviance, such as the mean squared error (MSE), mean absolute error (MAE), misclassification error, and AUC. MSE and MAE are not suitable for classification tasks due to their use for regression. Deviance is evaluated according to the log-likelihood error, and the AUC is the most popular evaluation criterion. Decreasing the deviance error is very important, as a small deviance represents good classification and optimization ability. Thus, we obtained the regression coefficients based on the smallest deviance. We also attempted to use the AUC criterion to select features, and the results changed little with deviance.

In the renal ultrasound image dataset, each patient had at least 9 image slices with different kidney representations and imaging views to facilitate renal status observation by radiologists. The combination of multiple slices was indeed beneficial for discriminating renal status. The classification performance comparison revealed that the accuracy and AUC of these four classifiers (logistic regression, SVM, random forest, and KNN) for patients were at least 5% better than those for individual slices (Additional file 1: Table 2). The reason is that multiple ultrasonic slices with different imaging views complement each other to aid in the final classification. We have therefore demonstrated that the integration of multiple ultrasonic slices can improve the final classification performance and contribute to the discrimination of nephropathies. In the future, exploration of new algorithms to utilize multiple ultrasonic slices will be a promising research topic.

In this study, we verified the feasibility of histological classification with ultrasound images and provided an alternative diagnostic technique. The technique is non-invasive and shows great promise with significant safety and efficacy benefits. Although the data in this study were of high quality and were meticulously collected, there were some limitations of the study. For example, it had a limited sample size and few clinical types and was a single-centre study. The limited sample size might have resulted in overfitted and overly optimistic results. The analyses were restricted to the most common subtypes (IgA nephropathy and MN) and thus did not reflect the clinical diversity, and the lack of sub-stratification of IgA nephropathy and MN limits the clinical applicability of the results. To enable broad clinical application of this approach and to improve the classification effectiveness, large-scale prospective multi-centre studies are needed to validate our results. In addition, considering the sample size of the patient cohort evaluated, the lack of an independent external test dataset may limit the generalizability of our results despite the variety of measures used in our study, such as cross-validation. This limitation might influence the applicability of our findings to other patient populations. Finally, the main aim of this study was to assess whether renal ultrasonography radiomics features could be used for the histologic classification of glomerulopathy; therefore, we evaluated this possibility through simple feature extraction and classification. In the future, we will also deploy a novel deep learning method to improve classification performance. Furthermore, the underlying bio-pathological changes associated with specific radiomics feature profiles of glomerulopathy subtypes need further investigation.

## Conclusions

In conclusion, radiomics signatures extracted from renal ultrasound images can help to differentiate IgA nephropathy from MN. Radiomics analysis, a non-invasive method, is helpful for histological classification of glomerulopathy. This study may have a clinical impact, providing an unprecedented opportunity to improve the diagnosis of nephropathy and decision support for nephropathy treatment. In the future, in-depth studies are needed to confirm these findings.

## Supplementary Information

**Additional file 1**. Additional results.

## Data Availability

The data that support this study will be provided upon request to the authors, only for academic research purposes and with the commitment to cite this work.

## Abbreviations

MN: Membranous nephropathy
LASSO: Least absolute shrinkage and selection operator
SVM: Support vector machine
ROC: Receiver operating characteristic
AUC: Area under ROC curve
CKD: Chronic kidney disease
CT: Computed tomography
MRI: Magnetic resonance imagin
PET: Positron emission tomography
eGFR: Estimated glomerular filtration rate
CKD-EPI: CKD-epidemiology collaboration
ROIs: Regions of interest
GLCM: Gray-level co-occurrence Matrix
GLRLM: Gray-level run-length matrix
KNN: K-nearest neighbours
SD: Standard deviation

## References

[1] Zhang L, Wang F, Wang L, Wang W, Liu B, Liu J, Chen M, He Q, Liao Y, Yu X, Chen N, Zhang JE, Hu Z, Liu F, Hong D, Ma L, Liu H, Zhou X, Chen J, Pan L, Chen W, Wang W, Li X, Wang H (2012). Prevalence of chronic kidney disease in China: a cross-sectional survey. Lancet.

[2] Hou JH, Zhu HX, Zhou ML, Xu F, Liang DD, Shao SJ, Liu Y, Liu ZH (2018). Changes in the spectrum of kidney diseases: an analysis of 40,759 biopsy-proven cases from 2003 to 2014 in China. Kidney Dis (Basel).

[3] KDIGO clinical practice guideline on glomerular diseases (public review draft), June 2020. https://kdigo.org/wp-content/uploads/2017/02/KDIGO-GN-GL-Public-Review-Draft_1-June-2020.pdf

[4] Luciano RL, Moeckel GW (2019). Update on the native kidney biopsy: core curriculum 2019. Am J Kidney Dis.

[5] O'Neill WC (2014). Renal relevant radiology: use of ultrasound in kidney disease and nephrology procedures. Clin J Am Soc Nephrol.

[6] Moghazi S, Jones E, Schroepple J, Arya K, McClellan W, Hennigar RA, O'Neill WC (2005). Correlation of renal histopathology with sonographic findings. Kidney Int.

[7] Lambin P, Rios-Velazquez E, Leijenaar R, Carvalho S, van Stiphout RG, Granton P, Zegers CM, Gillies R, Boellard R, Dekker A, Aerts HJ. Radiomics: extracting more information from medical images using advanced feature analysis. Eur J Cancer. 2012; 48(4):441–6. 10.1016/j.ejca.2011.11.036 PMC4533986 22257792

[8] Aerts HJWL, Velazquez ER, Leijenaar RTH, Parmar C, Grossmann P, Carvalho S, Carvalho S, Bussink J, Monshouwer R, Haibe-Kains B, Rietveld D, Hoebers F, Rietbergen MM, Leemans CR, Dekker A, Quackenbush J, Gillies RJ, Lambin P (2014). Decoding tumour phenotype by noninvasive imaging using a quantitative radiomics approach. Nat Commun.

[9] Gillies RJ, Kinahan PE, Hricak H (2016). Radiomics: Images Are More than Pictures. They Are Data Radiology.

[10] Liu Z, Wang S, Dong D, Wei J, Fang C, Zhou X, Sun K, Li L, Li B, Wang M, Tian J (2019). The applications of radiomics in precision diagnosis and treatment of oncology: opportunities and challenges. Theranostics.

[11] Wu MH, Chen CN, Chen KY, Ho MC, Tai HC, Wang YH, Chen A, Chang KJ (2016). Quantitative analysis of echogenicity for patients with thyroid nodules. Sci Rep.

[12] Guo Y, Hu Y, Qiao M, Wang Y, Yu J, Li J, Chang C (2018). Radiomics analysis on ultrasound for prediction biological behavior in breast invasive ductal carcinoma. Clin Breast Cancer.

[13] Levey AS, Stevens LA, Schmid CH, Zhang YL, Castro AF, Feldman HI, Kusek JW, Eggers P, Van Lente F, Greene T, Coresh J (2009). A new equation to estimate glomerular filtration rate. Ann Int Med.

[14] Haralick RM, Shanmugam K, Textural features for image classification. IEEE Trans Syst Man Cybernet. 1973;3(6):610–621.

[15] Galloway MM (1975). Texture analysis using gray level run lengths. Comput Graphics Image Process.

[16] Chu A, Sehgal CM, Greenleaf JF (1990). Use of gray value distribution of run lengths for texture analysis. Pattern Recogn Lett.

[17] Tibshirani R.J. Regression shrinkage and selection via the lasso. J R Stat Soc Ser B (Methodological);1996: 267–288.

[18] Floege J, Amann K (2016). Primary glomerulonephritides. Lancet.

[19] Li C, Yao Z, Zhu M, Lu B, Xu H (2017). Biopsy-free prediction of pathologic type of primary nephrotic syndrome using a machine learning algorithm. Kidney Blood Press Res.

[20] Beck LH, Bonegio RGB, Lambeau G, Beck DM, Powell DW, Cummins TD, Cummins JBK, David JS (2009). M-type phospholipase A2 receptor as target antigen in idiopathic membranous nephropathy. N Engl J Med.

[21] Floege J, Barbour SJ, Cattran DC, Hogan JJ, Nachman PH, Tang SCW, Wetzels JFM, Cheung M, Wheeler DC, Winkelmayer WC, Rovin BH, Conference Participants. Management and treatment of glomerular diseases (part 1): conclusions from a Kidney Disease: Improving Global Outcomes (KDIGO) Controversies Conference. Kidney international. 2019;95(2):268–280. 10.1016/j.kint.2018.10.018 30665568

[22] Uhlig J, Leha A, Delonge LM, Haack AM, Shuch B, Kim HS, Bremmer F, Trojan L, Lotz J, Uhlig A, Radiomic features and machine learning for the discrimination of renal tumor histological subtypes: a pragmatic study using clinical-routine computed tomography. Cancers (Basel). 2020;12(10):3010. 10.3390/cancers12103010 PMC7603020 33081400

