# Supplementary material for: Preliminary study on the application of renal ultrasonography radiomics in the classification of glomerulopathy
Source: BMC Med Imaging. 2021 Jul 23;21:115. doi: 10.1186/s12880-021-00647-8 (PMC8305820; doi:10.1186/s12880-021-00647-8)
Supplement: Supplementary file 1 — Additional file 1. Additional results. [file 12880_2021_647_MOESM1_ESM.docx]

**S1 Table. The classification performance of four classifiers on image slice and patient**

| **Model** | | **Logistic regression** | **SVM** | **Random forest** | **KNN** |
| --- | --- | --- | --- | --- | --- |
| **Accuracy** | **Test set (slice)** | 0.6144 | 0.6405 | 0.6275 | 0.5556 |
|  | **Test set (patient)** | **0.7647** | **0.7059** | **0.7059** | 0.5294 |
| **AUC** | **Test set (slice)** | 0.6415 | 0.6531 | 0.6849 | 0.5802 |
|  | **Test set (patient)** | **~~0.7361~~ 0.7500** | **0.7222** | **0.7639** | **0.7361** |

**S2 Table. The nephropathy classification performance of four classifiers on the selected features**

| **Model** | | **Logistic regression** | **SVM** | **Random forest** | **KNN** |
| --- | --- | --- | --- | --- | --- |
| **Accuracy** | **All features** | 0.7059 | 0.6471 | 0.7059 | 0.5294 |
|  | **Selected features** | **0.7647** | **0.7059** | 0.7059 | 0.5294 |
| **AUC** | **All features** | 0.7361 | 0.7222 | 0.7361 | 0.7292 |
|  | **Selected features** | **0.7500** | 0.7222 | **0.7639** | **0.7361** |
